# Government responses to COVID-19 and impact on GBV services and programmes: comparative analysis of the situation in South Africa, Kenya, Uganda, and Nigeria

**DOI:** 10.1080/26410397.2023.2168399

**Published:** 2023-03-01

**Authors:** Neetu A. John, Paul Bukuluki, Sara E. Casey, Dhruvi B. Chauhan, Moriam O. Jagun, Nicoletta Mabhena, Mary Mwangi, Terry McGovern

**Affiliations:** a Assistant Professor, Global Health Justice and Governance Program, Heilbrunn Department of Population and Family Health, Mailman School of Public Health, Columbia University, New York, NY, USA.; b Associate Professor, Makerere University, Kampala, Uganda; c MPH Student, Global Health Justice and Governance Program, Heilbrunn Department of Population and Family Health, Mailman School of Public Health, Columbia University, New York, NY, USA; d Consultant, Center for Bridging Health Gaps, Lagos State, Nigeria; e Managing Director, ResearchLinkME, Johannesburg, South Africa; f Independent Consultant, Nairobi, Kenya; g Director, Global Health Justice and Governance Program, Harriet and Robert H. Heilbrunn Professor and Chair, Heilbrunn Department of Population and Family Health, Mailman School of Public Health, Columbia University, New York, NY, USA

**Keywords:** COVID-19 policies, GBV prevention and response, sub-Saharan Africa, service disruption

## Abstract

As governments impose restrictive policies to contain infectious disease outbreaks, pre-existing gender-based inequalities are often exacerbated, increasing the risk of gender-based violence (GBV). Despite international guidance on the need for continued provision of GBV services during emergencies, governments often de-prioritise GBV services and programmes. We conducted a rapid assessment in South Africa, Kenya, Uganda, and Nigeria to examine the impact of COVID-19 policies on the availability of GBV prevention and response services. The study team interviewed 80 stakeholders representing different GBV services in the four countries. The interviews revealed strikingly similar government mis-steps that disrupted the availability of comprehensive GBV services. In all four countries, the government’s failure to exempt the provision of multi-sectoral GBV services from initial lockdown restrictions led to confusion and disrupted the provision of critical GBV services such as clinical management of rape, legal and judicial services, psychosocial services, availability of shelters, and community-based prevention activities. The government’s imposition of curfews, stay-at-home orders, and transportation restrictions further diminished access to services. Governments must strengthen currently available GBV prevention and response services and be better prepared for future pandemics. Following international guidelines, governments should deem GBV services as essential from the beginning with clear implementation plans. Governments must invest in community-based solutions and the expansion of digital tools to ensure everyone, especially those likely to be structurally excluded, have access to critical services during an emergency.

## Résumé

Lorsque les gouvernements imposent des politiques restrictives pour contenir les flambées de maladies infectieuses, les inégalités de genre préexistantes en sont souvent exacerbées, ce qui augmente le risque de violences sexistes. En dépit des recommandations internationales sur la nécessité d’assurer des services de prévention des violences sexistes pendant les urgences, les gouvernements dépriorisent souvent ces services et programmes. Nous avons réalisé une évaluation rapide en Afrique du Sud, au Kenya, au Nigéria et en Ouganda pour examiner les répercussions des politiques relatives à la COVID-19 sur la disponibilité des services de prévention et de traitement des violences sexistes. L’équipe de l’étude a interrogé 80 parties prenantes représentant différents services liés aux violences sexistes dans les quatre pays. Les entretiens ont révélé des faux-pas gouvernementaux étonnamment similaires qui ont compromis la disponibilité de services complets en matière de violences sexistes. Dans les quatre pays, l’incapacité du gouvernement d’exempter la prestation de services multisectoriels de violences sexistes des restrictions du confinement initial a suscité la confusion et a désorganisé le fonctionnement de services essentiels comme la gestion clinique du viol, les services juridiques et judiciaires, les services psychosociaux, la disponibilité d’abris et les activités de prévention communautaire. L’imposition par les autorités de couvre-feux, l’obligation de rester chez soi et les restrictions de transport ont encore diminué l’accès aux services. Les autorités gouvernementales doivent renforcer les services actuellement disponibles de prévention et de traitement des violences sexistes et être mieux préparés aux futures pandémies. D’après les directives internationales, les gouvernements devraient considérer les services de violence sexiste comme essentiels depuis le début, avec des plans de mise en œuvre clairs. Les pouvoirs publics doivent investir dans des solutions communautaires et l’expansion des outils numériques pour veiller à ce que tout le monde, en particulier les personnes qui risquent d’être structurellement exclues, ait accès à des services essentiels pendant une urgence.

## Resumen

A medida que los gobiernos imponen políticas restrictivas para contener los brotes de enfermedades infecciosas, a menudo se exacerban las desigualdades de género preexistentes, por lo cual aumenta el riesgo de violencia de género (VG). A pesar de la orientación internacional sobre la necesidad de la continua prestación de servicios relacionados con VG durante emergencias, los gobiernos a menudo despriorizan los servicios y programas de VG. Realizamos una evaluación rápida en Sudáfrica, Kenia, Uganda y Nigeria para examinar el impacto de las políticas de COVID-19 en la disponibilidad de servicios de prevención y respuesta a VG. El equipo de estudio entrevistó a 80 partes interesadas que representaban diferentes servicios de VG en los cuatro países. Las entrevistas revelaron tropiezos sorprendentemente similares del gobierno que interrumpieron la disponibilidad de servicios integrales de VG. En los cuatro países, el hecho de que el gobierno no eximiera la prestación de servicios de VG multisectoriales de las restricciones iniciales de cierre causó confusión e interrumpió la prestación de servicios de VG esenciales, tales como el manejo clínico de la violación, servicios jurídicos y judiciales, servicios psicosociales, disponibilidad de refugios, y actividades de prevención comunitarias. La imposición por el gobierno de toques de queda, órdenes de permanecer en casa y restricciones de transporte disminuyeron aun más el acceso a los servicios. Los gobiernos deben fortalecer los servicios de prevención y respuesta a la VG disponibles actualmente y estar mejor preparados para futuras pandemias. Siguiendo las directrices internacionales, los gobiernos deben considerar los servicios de VG como esenciales desde el principio con planes de ejecución precisos. Los gobiernos deben invertir en soluciones comunitarias y en la ampliación de herramientas digitales para garantizar que todas las personas, especialmente aquéllas más propensas a sufrir exclusión estructural, tengan acceso a servicios esenciales durante una emergencia.

## Background

### Overview

Pandemics present complex humanitarian crises with impacts that exacerbate pre-existing gender-based and other socio-economic vulnerabilities.^1–3^ Women, girls, and other structurally excluded populations such as sex workers and sexual and gender minorities are often hard hit during infectious disease outbreaks as governments impose restrictive policies to contain them without considering their unintended consequences.^4^ Gender-blind government policies may increase vulnerability to gender-based violence (GBV) as social networks and systems break down and survivors are trapped inside homes with their perpetrator.^5^ In periods of crisis, despite this heightened need, governments often de-prioritise GBV services and programmes and do not deem them as essential services. Survivors can face life-long health consequences such as unintended pregnancies, sexually transmitted infections, fistula, pelvic pain, urinary tract infections, poor mental health, and other chronic conditions due to lack of access to timely critical care.^6^ The Ebola outbreak in West Africa highlighted the negative impacts on women and girls of mobility restrictions, school closures, and de-prioritisation of GBV and sexual and reproductive health (SRH) services.^5^ GBV increased in the region during this prolonged emergency, while access to GBV services became harder. In the Democratic Republic of Congo, these policies and the environment of fear and isolation during the outbreak resulted in women and girls experiencing increased emotional violence in their homes, and delaying seeking post-rape care.^7^

Early reports suggest that the COVID-19 pandemic, like previous epidemics, has exacerbated gender-based inequalities with particularly severe impacts on harder to reach women and girls.^8–10^ Mirroring earlier pandemics, governments across the globe imposed restrictive policies to contain the pandemic that ignored gender-based vulnerabilities and did not initially deem GBV and SRH services as essential.^11^ Evidence suggests that in most regions of the world, GBV increased during the pandemic,^12–14^ while service providers in multiple countries struggled to provide care amidst insufficient resources, lockdowns and mobility restrictions, and de-prioritisation of GBV services in government emergency response plans.^15^

This rapid study investigated the impact of government policies to contain COVID-19 on the availability of comprehensive GBV prevention and response services in South Africa, Kenya, Uganda, and Nigeria during the first six months of the pandemic from March to September 2020. All four countries implemented their first pandemic-related lockdowns and movement restrictions in March 2020, without an explicit declaration that GBV prevention and/or response services would be considered essential and remain available. These countries only included GBV services in their essential services list after public outcry over increasing incidents of GBV surfaced, and their initial efforts were piecemeal and lacked sector-specific guidelines for implementation.^16^ In South Africa, President Cyril Ramaphosa declared on 13 April 2020 that GBV response services, including shelters, medico-legal services, and psychosocial support, were essential and would remain operational.^17^ In early April 2020, the Kenyan Ministry of Health released its guidelines for continuing care for sexual, reproductive, maternal, and newborn healthcare needs, which included clinical services needed by survivors of GBV, but other essential GBV services were not mentioned.^18^ In Uganda, the Minister of Gender, Labour and Social Development called upon all stakeholders in government, civil society, and the community to implement and enforce laws, policies, and programmes aimed at GBV prevention and response on 28 April 2020.^19^ In Nigeria, while health services were deemed essential, GBV services were not clearly designated as essential, and policies varied across states.^20^

As these restrictive policies were being implemented, available information from call centres and hotlines indicated a surge in GBV in these countries. In Kenya, calls to the national GBV hotline increased by 775% in March and April 2020,^21^ resulting in an additional 3650 cases of GBV reported from March through July.^22^ In South Africa, a national counselling hotline called Lifeline SA documented a 500% increase in the number of GBV calls in the two months after the lockdown began.^23^ In Nigeria, government-collected data from two-thirds of the states demonstrated a 149% increase in reports of GBV from March to April, 2020.^24^ In Uganda, 3280 cases of GBV were reported to police in April 2020 as compared to a monthly average of 1137 cases in 2019.^25^

Clearly, government policies in the early phase of the pandemic narrowly focused on infection control and did not consider the potential impact of the policies on historically marginalised and vulnerable groups such as women and girls. We provide in-depth analysis of the impact of these policies on availability and access to GBV services and programmes at a time of heightened need. For the purposes of this paper, we use the term GBV to indicate violence against women and girls.

### Pre-COVID GBV context

#### GBV prevalence

Even prior to COVID-19, GBV was highly prevalent in the four countries. Most recent pre-COVID estimates from the countries suggest that high levels of intimate partner violence (IPV) were prevalent. In South Africa, 26% of ever partnered women had experienced physical, sexual, or emotional IPV in their lifetime, while the corresponding figure for Kenya was 47%.^26,27^ In Uganda and Nigeria, 56% and 36% of ever partnered women respectively had experienced at least one form of IPV over their lifetime.^28,29^ Adolescent pregnancies and harmful practices such as child marriage and female genital mutilation also persist in these countries.^26–29^

#### Policy and legal framework

The four countries are State Parties to international and regional human rights conventions that prohibit GBV, although only Kenya and South Africa incorporated these treaties into national law.^30–33^ In all four countries, plural legal systems create contradictions and inconsistencies through the simultaneous application of statutory, customary, and religious laws (Table 1). For example, despite South Africa’s progressive constitution and laws, traditional courts, also referred to as chiefs’ courts, are an important part of the administration of justice in much of the country, especially in rural areas, where they administer customary law in over 80% of villages.^34^ Although civil and criminal law in Nigeria prohibits polygamy, Islamic and customary law permit the practice.^35^ Paying bride price, still a popular practice in these countries, is used to legitimise violence against women. Many Ugandans, for example, perceive bride price as indicating that a woman has been “bought” into the man’s household, reducing her decision-making role, limiting her independence, and perpetuating unequal gender power relations.^36^ All but Nigeria have standalone GBV policy frameworks or action plans that commit to a multi-sectoral approach to tackle GBV; however their operationalisation and adequate budgeting for coordinated services and programmes have remained a challenge.^37–40^

**Table 1.** Policy and legal frameworks related to GBV

	South Africa	Kenya	Uganda	Nigeria
**International human rights conventions**	State party to nearly all, incorporated in national law	State party to nearly all, incorporated in national law	State party to nearly all	State party to many, but most not domesticated
**Law against domestic violence**	*Domestic Violence Act* has expansive definition of domestic violence; Domestic Violence Amendment Bill included dating and customary relationships	*Protection Against Domestic Violence Act*, includes civil protective orders	*The Domestic Violence Act 2010*	No, except in Federal Capital Territory (Abuja) *Violence against Persons Prohibition (VAPP) Act*
**Marital rape**	Prohibited, but customary and religious law impacts prosecution	*Protection Against Domestic Violence Act* provides only civil, sanctions; Sexual Offenses Act excludes marital rape	Not addressed in relevant laws	Excluded from federal and many state penal codes
**Traditional practices**^a^**prohibited by law**	Yes, but traditional courts commonly permit them; polygamy legal	Yes, but prosecutions rare; polygamy legal	Yes, but widow inheritance, polygamy not explicitly outlawed	Some yes in some states, but many continue under customary law systems in many states; polygamy prohibited
**National GBV Policy or Action Plan**	National Strategic Plan on Gender-Based Violence and Femicide 2020–2030	National Policy for Prevention and Response to GBV	National Action Plan on Elimination of GBV	No, but some GBV goals included in the 2006 National Gender Policy

^a^ Traditional practices include child marriage, female genital mutilation, wife inheritance, virginity testing, and widow cleansing.

#### Service provision

Despite policy commitments as described previously, GBV services and programmes were underfunded and de-prioritised even prior to the pandemic. Governments across the four countries largely relied on bilateral and multilateral donors to fund GBV services and programmes. In South Africa, after brutal attacks against women and protests in 2019, the government prepared a plan and allocated funding to address violence against women, which was to be implemented in 2020. The governments use a mix of providers from the government sector to local non-governmental organisations (NGO) and international NGOs to deliver services and programmes. Effectively coordinating between the different sectors and providers has always been challenging, leading to fragmented services, breakdown of referral mechanisms, and variation in service quality based on type of provider and geography. Comprehensive multi-sectoral services with well-functioning referral systems were primarily available in major urban areas, while rural and remote areas had sub-optimal coverage.^41–43^ For example, in Kenya, comprehensive high-quality GBV response services are available in hospital-based One Stop Centres (OSCs) in large urban centres, while in rural areas, survivors lack access due to distance and transportation costs.^43^ In South Africa, OSCs are more widely available, at least at the provincial level.^44^ In Uganda, in the absence of investments in OSCs, the government relies on referral mechanisms that are managed by both government departments as well as NGOs providers, but these referral systems tend to be weak in rural and remote areas.^42^ In Nigeria, only 17 of 36 states have OSCs (Sexual Assault Referral Centres) that provide free clinical, counselling, and support services to GBV survivors.^45^ Additionally, despite commitments to multi-sectoral and comprehensive approaches to tackle GBV, de-prioritisation and lack of financial investments have led to a prioritisation of sexual violence and a focus on providing services such as clinical management of rape, over other forms of GBV such as domestic violence, physical and emotional violence, and services such as shelters, other psychosocial services, and prevention programmes. Shelters are primarily run by NGOs and are scarce in Kenya and Uganda, while South Africa has a more robust network of shelters managed both by the government and NGOs but still fell short of the demand for services.^43,44^ Despite investments in legal reform and building capacity of police and judicial officials, accessing justice remains a challenge due to corruption, lack of implementation of GBV laws, and inefficiencies of the judicial system.^46^ Although civil society in Uganda and South Africa have been at the forefront of investing in creative GBV prevention programmes, overall, prevention work remains underfunded across the four countries and does not systematically reach a large majority of the affected population. Due to limited funding, these programmes are often layered over HIV programmes and do not comprehensively address the varied drivers and groups affected by GBV.^47^

## Methods

We conducted a mixed-methods study which included in-depth interviews and online surveys with GBV and SRH stakeholders in the four countries. This manuscript focuses on data from the in-depth interviews conducted with 80 GBV stakeholders, including GBV programme managers, service providers, donors, and government officials (Table 2). Respondents were selected purposively based on their experience and leadership in each country’s GBV prevention and response and their knowledge of and experience with a range of services, including judicial and police services, shelters, prevention, and healthcare services. Efforts were made to ensure representation across types of organisations (local and international NGOs, donors, government), and geographies.

**Table 2.** Study participants

	South Africa	Kenya	Uganda	Nigeria
Local NGO	7	8	10	10
INGO	2	2	4	4
Donor	2	5	6	4
Government	2	7		2
**Total**	**13**	**22**	**20**	**20**

Interviews were conducted remotely (via phone, Zoom, or Skype) in July–September 2020, using semi-structured interview guides. Interviewers took detailed notes during the interview. Interviews were also audio-recorded with the respondent’s consent. Interviews were not transcribed. The detailed notes were finalised after reviewing the audio recordings. We utilised both inductive and deductive analysis to identify emerging themes. A data matrix was created to examine similar impacts and differing impacts, as well as innovations and adaptations within each service area across the four countries.

Verbal informed consent was obtained from all participants. Only study staff had access to the recordings and interviewer notes. The study was determined to be exempt by the Institutional Review Board of Columbia University.

## Results

Respondents from all four countries perceived that government determination of what constituted essential services, coupled with lockdowns and mobility restrictions, greatly impeded the provision of and access to comprehensive GBV prevention and response services. Our findings outline the impacts of these governments’ policies on critical GBV services and programmes: (1) health services, (2) psychosocial services, (3) shelters, (4) judicial and police services, (4) community-based prevention services, and (5) coordination among these sectors.

### Health services

All four countries deemed health services essential but GBV services appeared to be de-prioritised. Respondents cited factors that made it hard for service providers to deliver services and survivors to utilise services. Respondents in all four countries described how the government decision to shift health personnel to the COVID response led to fewer available providers to serve the specific medical needs of survivors, including medical forensic exams. In Kenya, health workers overburdened by COVID-19 cases de-prioritised clinical management of rape cases. Survivors who did reach health facilities were sometimes told to return later for care, which some respondents associated with an observed rise in unintended pregnancies. In South Africa, the Department of Health focused on keeping the large public community health centres open, while smaller community clinics closed entirely. As one respondent from a local NGO in South Africa summarised, “everything else that wasn’t COVID-related had to take a back seat.” Respondents in Uganda described how it became even more challenging to find appropriate medical personnel to complete forensic exams (this was a challenge even prior to COVID-19) that led to delays in survivors having access to care that would help them with their legal cases.

*“There was always difficulty in filling Police Form 3 [forensic exam documentation] in cases of sexual violence because most of the health centers were busy attending to COVID-19 patients or the officers were not available, particularly those qualified to fill the Police Form 3 under law. In most cases it was time to attend to COVID-19.”* (Local NGO provider, Uganda)

Additionally, curfews and restrictions on public transportation limited the ability of survivors to visit clinics and receive appropriate medical treatment in a timely manner. Respondents from the four countries described how restrictions on public transportation constrained survivors’ access to medical clinics, especially for those living in rural areas.

*“We also have challenges in terms of physical visits because some survivors are not able to access the center because of restriction in movement.”* (Clinical service provider, Nigeria)

### Psychosocial services

Across the four countries, respondents noted that lockdowns completely disrupted in-person psychosocial services for survivors just as the demand for these services was rising. Many service providers responded to these demands by adapting service provision through use of virtual platforms and other modalities, often without support or guidance from the government.

*“We never imagined having to respond in terms of this kind of work without being physically present especially in the beginning it was very, very tough … not only because we were not able to be on the ground, the regulations were really unclear around … .who, where, what, all of those things we deal on a day to day basis.”* (INGO provider, South Africa)

In Kenya and South Africa, respondents described providing counselling services remotely to survivors during the lockdowns. In Uganda, the police department established a new toll-free hotline after women’s rights organisations demanded remote psychological counselling services and general information about violence against women and children be made accessible to the community. Organisations in Nigeria maintained use of a 24-hour helpline, staffed by professional counsellors who worked and received calls from their respective homes.

*“What happened was that during the lockdown, we had a child Helpline … . So what we did was that we've got our professional counselors taking the phones home so that they will be available 24/7, the work from the phone will be available 24/7, she received calls.”* (Local NGO provider, Nigeria)

While innovative, many respondents feared these virtual counselling services have limited reach, especially for structurally excluded populations such as adolescent girls and young women because in their setting these sub-populations often found it difficult to access phones or other virtual technology in a confidential and comfortable way.

*“The majority of survivors are more comfortable with one-on-one counselling. We serve [an informal settlement]. Some do not have access to phones. Survivors may not have airtime and some may be using other people’s phones.”* (Local NGO provider, Kenya)

To address these challenges, some organisations in Kenya experimented with utilising Community Health Volunteers (CHVs) to continue providing services to survivors. CHVs were trained to provide psychological first aid in-person during the lockdown and helped refer survivors to health facilities. The approach was successful in serving some harder to reach women, but some respondents feared negative perceptions of services provided by CHVs could hurt service provision in the future.

*“We did basic Community Health Volunteer training on basic psychological first aid for survivors before they refer cases to health facilities. The challenge is that clients will not believe that a CHV can give professional counselling.”* (Government official, Kenya)

### Shelter services

Respondents described stark differences across the four countries in shelter availability during lockdown, citing a mix of factors including government policies, existence of national champions, and pre-pandemic availability of shelters, and prevalence of private vs. public shelters.

In South Africa and Nigeria, where stronger networks of shelters existed, respondents noted that shelter services remained operational due to the advocacy and ingenuity of civil society organisations and government officials, among them South Africa’s National Shelter Movement and Nigeria’s Minister of Women Affairs. Although some organisations struggled to have enough space for survivors in shelters, respondents in Nigeria noted that it was helpful that a government official prioritised shelters and understood the needs of GBV survivors.

*“It was very encouraging; the Minister of Women Affairs was right at the forefront. It was evident from what she said that she was current with the news that COVID-19 was associated with increased cases of GBV in France, Germany and one particular case in Lagos which really got to her. She then ensured she touched base with us on a weekly basis to know what was going on with the shelter, the number of cases reported as well as needs of survivors and ways they could support such as raising awareness for the needs of rapid response.”* (INGO provider, Nigeria)

In South Africa, the National Shelter Movement as an organisation was proactive in ensuring shelter availability during the COVID-19 outbreak. Partnerships were established with Uber and other private transportation services to ensure survivors had transportation options to access the shelters. Shelters were perceived to be safely run with few COVID outbreaks at the beginning of the pandemic.

In Kenya, a country with shortage of shelters, shelter availability in the country further suffered when the lockdown was imposed. The lack of state-funded shelters contributed to a gap in protecting women and girls during the pandemic. After announcement of the Government's COVID-19 containment measures, many shelters closed completely. The shelters that remained open were discouraged from taking in new residents by the government’s imposition of medical documentation certifying new residents were COVID-19 free, which was hard to obtain.

*“Most of the shelters are privately owned and when COVID hit, they sent survivors back home because they could not afford to keep them and the ones [shelters] open are not admitting people. Most are supported by individuals.”* (Local NGO provider, Kenya)

*“If you rescue a survivor, they must have a COVID-19 certificate but it takes 14 days; this is a challenge for us.”* (Government official, Kenya)

In all four countries, transportation restrictions made shelters harder to access. While all survivors were affected by these restrictions, many respondents noted that women and girls living in rural and remote areas were harder hit due to limited public transportation options and lack of shelters in these locations. In South Africa, organisations partnered with Uber to ensure women had safe transportation to shelters.

*“… remember when they said no more movements, it was very difficult for women in rural areas to access a shelter because remember motorcycles and public transport were banned … overall to be honest all survivors found it difficult to access services and shelters at that time because remember there was no exemption at that time so we could not do anything unless for those ones who lived near the shelter.”* (Local NGO provider, Uganda)

Respondents also described the challenges shelters faced in adhering to COVID-19 protocols while simultaneously handling the increases in demand. In Nigeria, some shelter organisations faced financial hurdles adapting to the COVID-19 social distancing protocols, as they had to independently find additional places for survivors and their children or even shelter staff. In Kenya, as demand increased, privately-run shelters struggled to take in additional survivors and worried about not having enough food and medicines for those in their care.

*“We didn’t have enough shelter to take up the capacity of women coming in at that time, we usually have a capacity of 20 at a time and we had to go way less than that if they had children … We had to bring in additional programmes on board, create emergency housing, book hotel accommodation to stay at least for some days which is outside what we’d normally do which was an additional expense we had to cover. I mentioned that we had to go out every weekend to give out food and medication to women.”* (Local NGO provider, Nigeria)

*“At the beginning, shelter worked well or there were few victims. Now they are full and lack support in equipment and food to feed victims … ”* (Government official, Kenya)

### Access to justice

#### Judicial services

According to respondents across the four countries, the governments shut down the courts either completely or partially, and/or GBV cases were de-prioritised, with other criminal cases taking precedence.

*“The courts of law again weren’t considered an essential service, so they also went into lockdown unfortunately, and when they remembered that, oh they need to try the ones who are breaking curfew and whatever, they pulled them back. But now their priorities were the ones who have faulted the Covid-19 guidelines and other crimes … now the GBV cases weren’t prioritised.”* (INGO provider, Uganda)

In Kenya, only GBV cases involving rape were considered essential; all other GBV-related cases were turned away from court hearings and were instructed to be dealt with at the level of the police, as one court official describes:

*“I used to handle 30 cases in a day, now it’s 2–3 cases in a day and what we are doing is just giving dates (for hearings).”* (Government official, Kenya)

In South Africa, courts remained open with reduced staff and in the early weeks largely limited the GBV cases they accepted to those involving a high level of physical violence. This ad hoc process meant that GBV cases the court officials judged not urgent were overlooked, sometimes with harsh consequences.

*“The courts were only processing protection orders for women who suffered physical violence, they wanted to see blood.”* (Local NGO provider, South Africa)

*“[One woman] was turned away and she went home and then she and her children were killed by her partner.”* (Local NGO provider, South Africa)

Many respondents noted that although across the countries the courts eventually started providing virtual services during the lockdown, for many survivors, access to judicial services continued to be challenging in the absence of supportive resources, especially for survivors with limited resources and lack of digital literacy.

*“… some [women] don’t know how to operate Zoom or [Microsoft] Teams or a computer, especially for women in remote areas.”* (Local NGO provider, Kenya)

#### Police services

Respondents from the four countries noted that while police services were deemed essential, the police primarily focused on monitoring compliance to COVID-19 protocols and de-prioritised GBV cases. Respondents further noted that in many instances police impeded the ability of providers to provide services and survivors to access services. In the words of a respondent from South Africa:

*“Police hid behind COVID, giving permits and everything and not attending to issues of crime and violence against women and girls.”* (Local NGO provider, South Africa)

Across the countries, GBV survivors also found it hard to access police due to fewer staff at each site, curfews and mobility restrictions, lack of personal protective equipment to enter police stations, and being trapped with their perpetrator. In Kenya, respondents said survivors were afraid to report cases to the police outside of curfew hours due to fear of being harassed or mistreated by police for breaking curfew. Respondents working with female sex workers in Kenya particularly noted police use of curfew violations to harass sex workers who continued to work. Survivors who did access police stations found that police desks specific to gender issues were understaffed as staff were often redeployed to tackle COVID enforcement. Similarly, in Uganda, the critical police division for reporting GBV cases, the Child and Family Protection Unit, was not deemed essential.

*“The major problem is that we have police involved in curfew issues. Most Gender Units are understaffed to respond to GBV … At night victims fear going to the police station due to curfew hours, and people are not supposed to be outside.”* (Government official, Kenya)

### Community-based prevention activities

According to respondents, community-based GBV prevention activities came to a halt in the first few weeks of the pandemic following government-mandated lockdown policies. Under these circumstances, organisations had to either stop functioning or identify different service provision modalities, which were not always easy, especially without clear guidance or support from the government. With growing need for services, organisations in the different countries pivoted to provide services via virtual platforms, with varying levels of success. Respondents in Uganda described increasingly utilising web platforms like Zoom or Skype to organise and implement their prevention interventions virtually. However, many organisations faced challenges adapting to virtual platforms. For example, organisations with prevention service models that relied on integrated community engagement struggled to plan and adapt quickly, and some were unable to continue delivering services in a new way. As mentioned previously with virtual counselling services, respondents expressed concern that virtual prevention activities did not reach many who lacked access to phones or technology.

*“Just like anyone else, the lockdown directives just came without giving us time to plan. And then it came with very quick changes of what needed to happen in the community and yet a lot of our work happens in the community. That meant a lot of things and our GBV prevention and response programmes could not continue normally.”* (INGO provider, Uganda)

In all four countries, implementing COVID-19 protocols increased the cost of delivering services, with the shift to remote work requiring internet connectivity at home, computers, and other equipment. In Kenya, small community sessions gradually resumed in May 2020, but organisations still faced challenges implementing these interventions effectively, with little support from the government, including having to acquire special permits for events, adjusting typical travel protocols, and generally adhering to the COVID-19 guidelines.

*“We need permits from the (police) commander to do what was previously easy to do. You need transport to carry no more than seven people. Getting venues that are aligned to MOH guidelines. These have been the challenges.”* (Local NGO provider, Kenya)

### Collaboration opportunities across GBV sectors

In all four countries, pre-pandemic referral systems depended on GBV providers collaborating and coordinating across sectors to provide a full range of services, with varying success. According to respondents, the pandemic’s increased demand for services galvanised efforts to provide coordinated services and care. In South Africa, respondents reported that their collaborations improved and that relationship-building with other GBV organisations accelerated during the pandemic. Smaller local organisations found more ways to connect with larger NGOs and private foundations during the crisis. This collaboration and the use of virtual platforms led to more partnerships.

*“I have got partners now that I have never had before.”* (Local NGO provider, South Africa)

*“What has helped us to continue, I think, has been partnering with many organisations and trying to support locally based organisations because they then were facing the brunt of the issues.”* (INGO provider, South Africa)

GBV stakeholders in different sectors in Kenya reported working more closely together than before, with more support and recognition from the government as awareness grew about the scope of GBV during the crisis. In Nigeria, some respondents thought that the shift to using remote platforms for collaborative stakeholder meetings allowed younger organisations to engage more with different GBV partners than when meetings were in person, and increased networking improved these younger organisations’ access to funding opportunities. Improved opportunities to collaborate were highlighted as a positive outcome across the countries.

## Discussion

Our study suggests that in all four countries, the government’s initial response to the COVID-19 pandemic was characterised by similar policy mis-steps that ignored GBV services and programmes and impeded the availability of comprehensive GBV prevention and response services at a time of heightened need for women and girls. According to respondents from the four countries, the initial exclusion of GBV from the respective governments’ essential services list delayed guidance and allocation of resources that were needed for continued provision of comprehensive GBV prevention and response services and created confusion among service providers. Restrictive polices such as curfews, transportation and movement restrictions, and social distancing protocols further compounded both availability and access to services.

The study also indicates that even if a sector relevant for GBV services was deemed essential, GBV cases were de-prioritised, and no arrangements were made to ensure access and continuity of services to survivors. For example, although health services were deemed essential in all countries, clinical management of GBV was de-prioritised and confusion persisted over its status. In the initial weeks and months of restriction, GBV-specific activities were ignored in guidance. Health systems made no provisions to ensure survivors could access medical services during periods of mobility restrictions, and often re-assigned staff from GBV services to COVID-19 care. Similarly, while police services were deemed essential, services for GBV survivors were not prioritised. Policing in all four countries focused on ensuring compliance to COVID-19 containment policies, and police officers responsible for GBV support services were often redeployed to that end. GBV health providers and survivors reported abuse and harassment by police for being out after curfew, suggesting that police were not aware that some GBV services were deemed essential.

Other critical components of a comprehensive GBV response such as judicial, psychosocial, and shelter services were not always deemed essential at the beginning of the pandemic, and these services were disrupted, causing confusion and distress. Judicial services largely ceased functioning when the initial pandemic restrictions were imposed. As increasing GBV incidence demonstrated need for judicial services, governments in each country made piecemeal efforts to restore services using different modalities. Courts began using remote platforms to continue court hearings using online and mobile technologies but these shifts to alternative platforms were slow. Lack of access to and familiarity with digital tools discouraged many survivors from accessing courts. Psychosocial services also halted as restrictions were imposed, and providers had to quickly adapt to remote service provision as demand for services grew. Both government and NGO providers began successfully using toll-free lines and other virtual platforms to provide counselling and other support services. However, many NGOs felt ill-prepared for the transition, due to funding shortages and lack of appropriate government guidance, and felt overwhelmed by demand for services. In addition, many women, especially girls and young women, were unable to safely use virtual platforms as they lacked telephones or internet access. Availability of shelter services varied across the countries based on civil society or government leadership, as well as availability of shelters prior to the pandemic. South Africa and Nigeria were relatively more successful in ensuring availability of shelters due to a stronger network of shelters, and civil society and government champions. Across the four countries, however, restrictive policies and limited availability of public transportation meant that shelters were harder to access, particularly for those living in remote areas. Shelters also lacked the physical and financial resources to keep up with the increased demand for services.

Study findings suggest that community-based prevention activities were hardest hit by the restrictive policies and came to a halt in all four countries in the beginning of the pandemic. As the need for prevention activities become clear, many providers tried to adapt programme activities using virtual platforms. However, some found it hard to absorb the financial cost of remote platforms, and others felt that remote platforms were not appropriate for the kind of programming they were engaged in. Many also realised that these modalities did not work for many of their beneficiaries as they lacked digital literacy and/or could not access technology. To improve the reach of their programmes, some organisations successfully utilised community health workers to continue providing services to those hardest to reach, highlighting the critical need to build capacity of these providers as they are most likely to be successful in reaching structurally excluded populations during times of crisis.

The humanitarian community had previously identified the gendered impacts of pandemics from their experience responding to the Ebola epidemics in West Africa and the Democratic Republic of the Congo.^48,49^ Lessons learned from humanitarian response have been incorporated into key international guidance on Disaster Risk Reduction and Emergency Preparedness which identifies GBV as a critical aspect of strengthening individual and community resilience.^50^ Governments should ensure their emergency response plans comply with internationally recognised Minimum Standards for Prevention and Response to GBV in Emergencies.^51^ Women and girls, including women-led organisations, should be involved in emergency planning so that gendered dimensions of response plans are considered. Governments and donors should earmark funds for comprehensive GBV services and programmes in all emergency response plans. The plans should clearly outline how different components of a comprehensive GBV response, including health, judicial, shelters and psychosocial services, and referral systems, will remain operational amidst stay-at-home orders and other challenges. Anticipating a rise in GBV due to pandemic restrictions and related economic distress, GBV prevention activities should be allowed to continue, and other risk mitigation strategies such as providing cash and food transfers should be implemented.^52^ Further research is needed to understand why all four governments failed to adequately integrate gender into their national preparedness plans to ensure that GBV services were deemed essential as they rolled out their initial COVID-19 containment policies.

GBV experts in the humanitarian community, including the GBV Area of Responsibility and the Inter-Agency Working Group on Reproductive Health in Crises, quickly released guidance to mitigate GBV risks within COVID-19 response.^53,54^ Guidance from the humanitarian community included suggestions for non-specialist GBV actors to assess GBV-specific risks related to COVID-19 response in their sector to ensure continued access to coordinated services.^55^ In areas with active SRH and GBV sub-clusters in the humanitarian response, GBV programmes adapted more quickly than national actors to ensure GBV services continued. For example, safe spaces for women and girls in Dadaab and Kakuma refugee camps remained largely operational with adaptations to take into account social distancing by reducing group sizes and staggering times for activities.^56^ One strategy implemented in north-east Nigeria converted safe spaces into tele-health centres with individual phone booths, respecting infection prevention protocols, that women could use to contact GBV case managers.^57^ In Uganda, UNHCR established a call centre with trained counsellors speaking 15 languages to manage a helpline for GBV survivors.^58^ Overall, funding did not increase, and in some cases, was shifted to COVID efforts, preventing organisations from scaling up to meet the increased demand.^56^ Despite the learning and efforts of the humanitarian sector, the international community largely failed to put recommendations into practice when COVID-19 hit.^56,59^

To our knowledge, our study is among the first to comprehensively document how COVID-19 containment policies impacted availability and access to multi-sectoral GBV prevention and response services across four African countries in the beginning of the pandemic. We collected rich qualitative data from 80 GBV stakeholders representing service providers, programme managers, and government officials across different GBV sectors. The study has limitations that should be considered when interpreting results. Interviews were conducted with a convenience sample of GBV stakeholders. Although multiple efforts were made to contact key stakeholders, it is possible that those who were highly impacted (and very busy) were unable to respond. Similarity, while we tried to get representation across geographies, many respondents lived in the capital or other major cities. We were unable to secure interviews with government officials in all the four countries, which further limits the interpretability of our results. We also wanted to acknowledge that while this did not impact the quality of our findings, we decided not to seek local IRB review in each of the countries and only sought IRB approval at the global level because of the nature and goals of our study. Our study was conceptualised as a technical consultation with GBV and SRH stakeholders to provide rapid usable information about the state of GBV and SRH services to advocacy groups on the ground. After consulting with in-country partners, we concluded that going through local IRB review processes in the middle of the COVID-19 pandemic would unnecessarily delay the consultations and reduce the value of the material for groups on the ground. At the global level, Columbia University’s IRB declared the study as exempt research as participants were speaking in their professional capacity and drawing on their knowledge and experience of working in the GBV/SRH spaces and were not being asked to share personal information or personal data. Despite the exemption, we obtained informed consent from all participants, and all data were de-identified to ensure confidentiality and privacy of participants.

Our study suggests that the COVID-19 pandemic put tremendous stress on already weak GBV prevention and response systems in the four countries. Hence, beyond pandemics, greater national and donor investments in GBV prevention and response services are greatly needed to strengthen fragmented GBV prevention and response systems in the countries and ensure their sustainability. Fully implementing existing GBV legislation and policies and addressing gaps in legal and policy frameworks, giving greater leadership roles to GBV providers in policy design and implementation, strengthening and expanding multi-sectoral services, and establishing effective coordination between sectors, can greatly improve the quality and availability of GBV services. Governments and development actors should learn from the ability of humanitarian actors to transition quickly. Investments in gender transformative interventions and women’s empowerment are also needed to tackle the root causes of GBV and prevent it from occurring.^60^ The pandemic has also highlighted the potential and limits of technology for continuing service provision given the digital divide, and the critical importance of building community-based platforms for reaching structurally excluded populations. Countries should continue to invest in community-based institutions and make concerted efforts to expand digital literacy and access to digital tools to ensure that critical services are available to everyone, even those hardest to reach.
